# Pathogenic Actions of Cell Adhesion Molecule 1 in Pulmonary Emphysema and Atopic Dermatitis

**DOI:** 10.3389/fcell.2015.00075

**Published:** 2015-11-20

**Authors:** Azusa Yoneshige, Man Hagiyama, Mitsugu Fujita, Akihiko Ito

**Affiliations:** ^1^Department of Pathology, Faculty of Medicine, Kinki UniversityOsaka, Japan; ^2^Department of Microbiology, Faculty of Medicine, Kinki UniversityOsaka, Japan

**Keywords:** degeneration, apoptosis, protease imbalance, ectodomain shedding, neuro-immune interaction

## Abstract

Cell adhesion mediated by adhesion molecules is of central importance in the maintenance of tissue homeostasis. Therefore, altered expression of adhesion molecules leads to the development of various tissue disorders involving cell activation, degeneration, and apoptosis. Nevertheless, it still remains unclear what initiates the altered expression of adhesion molecules and how the subsequent pathological cascades proceed. In this regard, cell adhesion molecule 1 (CADM1) is one of the candidates that is involved in the development of pathological lesions; it is an intercellular adhesion molecule that is expressed in various types of cells such as pulmonary cells, neurons, and mast cells. Recent studies have revealed that alterations in the transcriptional or post-transcriptional expressions of CADM1 correlate with the pathogenesis of pulmonary diseases and allergic diseases. In this review, we specifically focus on how CADM1 is involved in the development of pathological lesions in pulmonary emphysema and atopic dermatitis.

## Function of CADM1 under normal conditions

In the last two or three decades, a line of studies have revealed the molecular basis of cell adhesion to neighboring cells and surrounding extracellular matrices in our body. That is, cell adhesion is of crucial importance in human body homeostasis (Macara et al., 2014). Dedicated proteins directly mediating cell adhesion have been revealed such as cadherins, integrins, selectins, and immunoglobulin superfamily members; they are now called “adhesion molecules” (Coombe and Dye, 2010).

In particular, accumulating data demonstrate the importance of cell adhesion molecule 1 (CADM1), which is an intercellular adhesion molecule that belongs to the immunoglobulin (Ig) superfamily (Kuramochi et al., 2001). CADM1 comprises three major functional domains: an extracellular Ig-like domain, a single transmembrane region, and a short carboxy-terminal intracytoplasmic tail that is further composed of a protein 4.1-binding motif and a PDZ type II domain-binding motif (Kuramochi et al., 2001). Historically, CADM1 has had several nomenclatures such as spermatogenic immunoglobulin superfamily (SgIGSF), tumor suppressor in lung cancer 1 (TSLC1), nectin-like molecule 2 (Necl2), and synaptic cell adhesion molecule (SynCAM), based on its diverse functional nature in various cell lineages such as spermatogonia, lung epithelial cells, and neurons (Watabe et al., 2003). We have previously demonstrated that CADM1 is expressed in mast cells (Ito et al., 2003), biliary cells (Ito et al., 2007), pancreatic endocrine cells (Koma et al., 2008), and osteoblasts (Inoue et al., 2013). In addition, CADM1 has been shown to have several splicing isoforms; these isoforms expressed on the cell surface are named SP1 to SP4 based on the difference in the length of the juxtamembranous extracellular region (Biederer, 2006). Lung epithelial cells and mast cells exclusively express the SP4 isoform whereas neurons express various combinations of these four isoforms (Ito et al., 2003; Sakurai-Yageta et al., 2009; Hagiyama et al., 2011).

In epithelia, CADM1 is located on the lateral cell membrane and mediates neighboring cell adhesion via *trans*-homophilic interaction (Koma et al., 2008; Ito et al., 2012). CADM1 directly interacts with a spectrin-actin-binding protein DAL1 through the intracytoplasmic protein 4.1-binding motif to promote the formation of epithelial cell morphology (Yageta et al., 2002; Sakurai-Yageta et al., 2009). CADM1 also interacts with a membrane-associated guanylate kinase PALS2 through the PDZ type II domain-binding motif; this interaction controls epithelial cell polarity (Shingai et al., 2003; Wu et al., 2007). In mast cells, CADM1 mediates their adhesion to fibroblasts (Ito et al., 2003), airway smooth muscle cells (Moiseeva et al., 2013), and neurons (Furuno et al., 2005). In particular, CADM1 plays a central role in the direct cell-to-cell contact between mast cells and unmyelinated C-fibers of sensory neurons through *trans*-homophilical interaction to promote neurno-immune interaction between them (Furuno et al., 2005).

## Pathogenic actions of CADM1 in pulmonary emphysema

Pathologically, the adhesion molecules is involved in the development and progression of diseases and disorders such as tumors (Demetriou and Cress, 2004), inflammatory diseases (Macauley et al., 2014), and neurological diseases (Berezin et al., 2014). Then, how can they be pathogenic without genetic mutations? One of possible mechanisms is their susceptibility to proteolysis. Since the adhesion molecules are expressed on the cell membrane, they often undergo extra- and inter-membraneous enzymatic cleavage; this phenomenon is termed “ectodomain shedding” (Edwards et al., 2008). CADM1 is shed at the extracellular domain by a disintegrin and metalloproteinase 10 (ADAM10) protease to produce a membrane-bound α C-terminal fragment (αCTF); CADM1 is also shed by yet unidentified proteases to produce βCTF (Nagara et al., 2012; Mimae et al., 2014). Tobacco smoking is known to induce an imbalanced increase in protease activity within lung alveoli (Taraseviciene-Stewart and Voelkel, 2008). As a consequence, lung epithelial CADM1 is shed in smokers’ lungs at high levels to produce a substantial amount of αCTF and βCTF. Then, αCTF enters into the lung epithelial cytoplasm, accumulates in mitochondria, and depolarizes the mitochondrial outer membrane potential; this process results in mitochondrial apoptotic pathway (Mimae et al., 2014). Because the full-length CADM1 plays an important role in the maintenance of epithelial polarity (Sakurai-Yageta et al., 2009), an increase in CADM1 shedding with an accompanying decrease in the full-length CADM1 induces the disruption of alveolar cell polarity and the cell apoptosis (Yoneshige et al., 2015).

**Figure 1:**
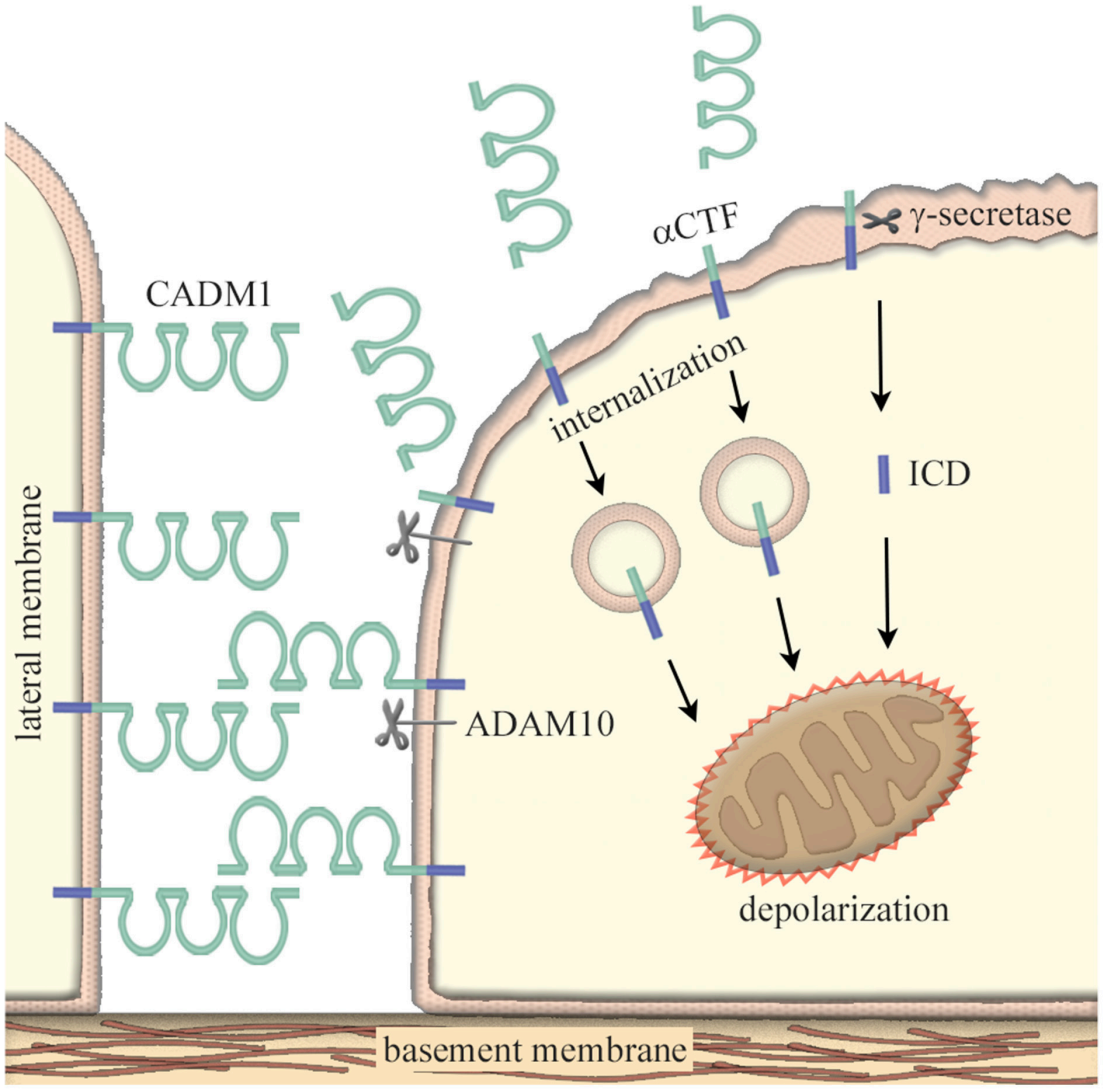
**Increased ectodomain shedding of CADM1 as a cause of epithelial cell apoptosis**. In epithelia, CADM1 is located on the lateral cell membrane and mediates neighboring cell adhesion via *trans*-homophilic binding. Under pathological conditions, ectodomain shedding of CADM1 is induced through an imbalanced increase in protease activity such as neutrophil elastases, macrophage matrix metalloproteinases, and/or the inactivation of anti-protease α1-antitrypsin. CADM1 is shed at the extracellular domain by a disintegrin and metalloproteinase 10 (ADAM10) protease to produce a membrane-bound α C-terminal fragment (αCTF). Then, the truncated product αCTF enters into cytoplasm, accumulates in mitochondria, and depolarizes the mitochondrial outer membrane potential; this process results in mitochondrial apoptotic pathway. αCTF can be further cleaved within its transmembrane region by γ-secretase to produce free fragments of the intracellular domain (ICD). In similar to αCTF, ICD moves into mitochondria and induces apoptosis of lung epithelial cells. An increase in CADM1 shedding with an accompanying decrease in the full-length CADM1 induces the disruption of alveolar cell polarity and the cell apoptosis.

There is the third CADM1 shedding event; the truncated products αCTF and βCTF can be further cleaved within its transmembrane region by γ-secretase to produce free fragments of the intracellular domain (ICD; Nagara et al., 2012). In a similar fashion to αCTF, ICD moves into mitochondria and induces apoptosis of lung epithelial cells (Hagiyama et al., 2015). Through these events, CADM1 is involved in the development and progression of pulmonary emphysema, a smoking-related lung degenerative disease characterized by the peripheral airspace enlargement associated with increased apoptosis of alveolar cells (Mimae et al., 2014; Figure 1). Similarly, CADM1-mediated pathological mechanisms have been proven in idiopathic interstitial pneumonia by inducing the apoptosis of type 2 alveolar cells (Yoneshige et al., 2015) and type 2 diabetic pancreas by inducing the apoptosis of insulin-secreting islet β cells (Inoue et al., 2014).

## Pathogenic actions of CADM1 in atopic dermatitis

We have also demonstrated pathological roles of CADM1 in atopic dermatitis (Hagiyama et al., 2013). It has long been known that pathological cell adhesion is observed between nerve fibers and mast cells in atopic dermatitis lesions (Jarvikallio et al., 2003). Here, mast cells are immune cells featured by cytoplasmic granules containing various bioactive molecules. Their unique characteristics is that, unlike other immune cells, they exist in the digestive and respiratory mucosa and skin dermis at a certain cell density under healthy conditions (Kitamura et al., 2007). Under pathological conditions, however, the number of mast cells is increased through the proliferation of tissue-resident mast cells and also the recruitment from circulating myeloid progenitor cells (Jung and Scholz, 2008).

**Figure 2:**
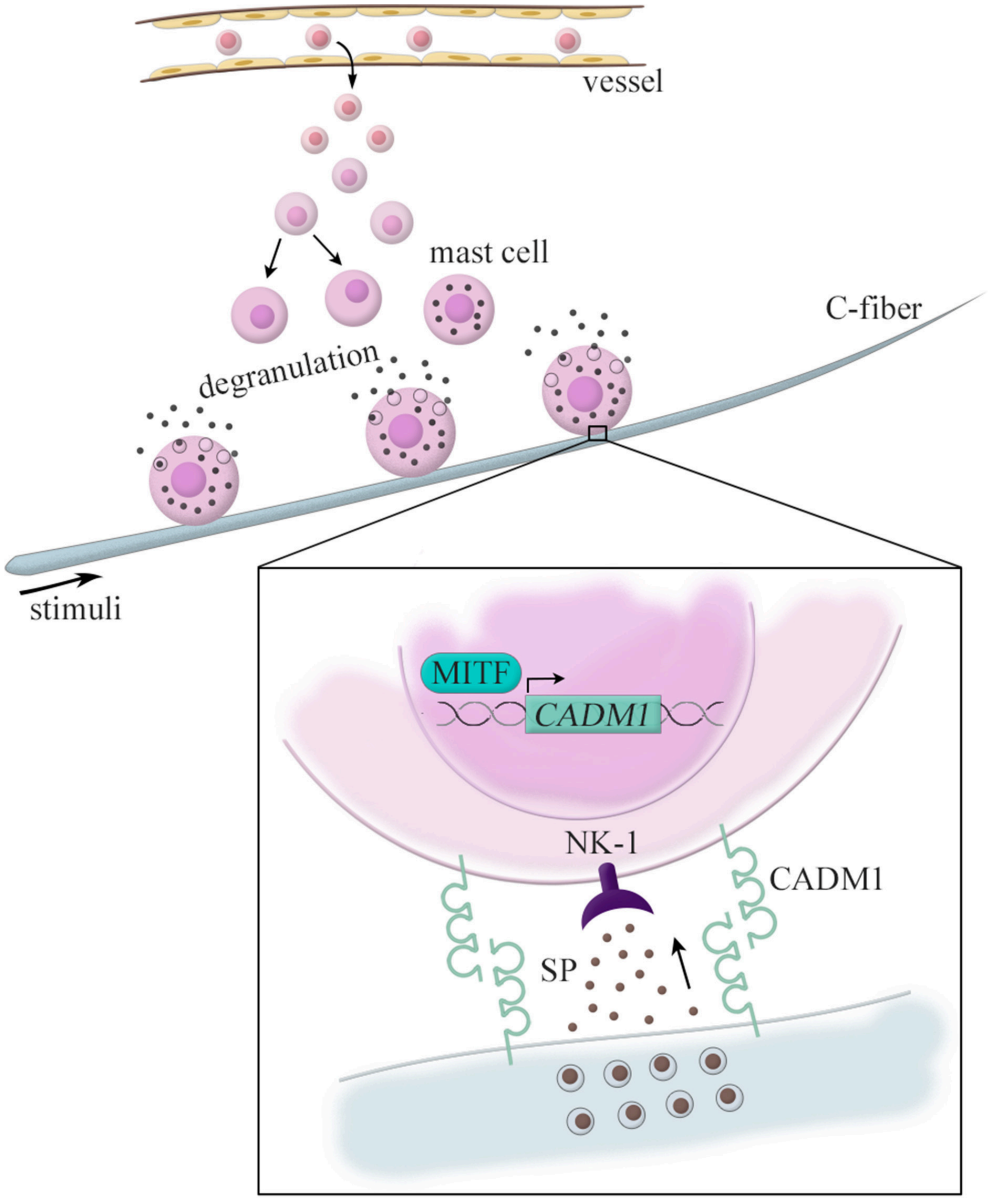
**CADM1-mediated nerve–mast cell interaction in the atopic dermatitis skin lesion**. Dermal mast cells are observed in contact with nerve fibers in the atopic dermatitis. These lesional mast cells express CADM1 at higher levels than those in the normal skin, which is induced by the basic helix-loop-helix type *microphthalmia*-transcription factor (MITF). CADM1-expressing mast cells interact with neurons to secrete substance P (SP), and the neuron-derived substance P further interacts with the cognate receptor neurokinin 1 (NK-1) expressed on the mast cells to induce their degranulation.

In a mouse model of hapten-induced atopic dermatitis, dermal mast cells were observed in contact with nerve fibers at high frequencies (Shiohara et al., 2004). Our laser capture microdissection-based RT-qPCT analyses revealed that the lesional mast cells expressed CADM1 mRNA at approximately five-fold higher levels than those in the normal skin (Hagiyama et al., 2013). This altered CADM1 expression appears to be induced by the basic helix-loop-helix type *microphthalmia*-transcription factor (MITF), a key factor for mast cell differentiation (Ito et al., 2003). When neurons and mast cells are co-cultured, the kinetic strength (force-induced dissociation rate) of mast cell adhesion to nerve fibers varies dependently on the expression levels of CADM1 on mast cells (Hagiyama et al., 2013). We have previously shown that the CADM1-expressing mast cells interact with neurons to secrete substance P and that such neuron-derived substance P further interacts with the cognate receptor neurokinin that is 1 expressed on the mast cells to induce their degranulation (Furuno et al., 2004, 2005). Taken together, these data suggest that neuron-mast cell adhesion (so called “neuro-immune interaction”) might be strengthened in atopic dermatitis through an increased expression of CADM1 in mast cells. This mechanism is recently highlighted as a molecular basis that can explain why mental stress exacerbates atopic dermatitis (Figure 2).

## Concluding remarks

As reviewed here, CADM1 is involved in the development of pulmonary emphysema through its ectodomain shedding in lung epithelial cells by promoting the apoptosis of lung epithelial cells. Since apoptotic cell death is frequently observed in various degenerative diseases, future studies may identify the protease-mediated shedding of adhesion molecules as a common cause for tissue degeneration. Furthermore, an increased expression of CADM1 is involved in the development of atopic dermatitis by promoting the neuro-immune interaction. Recent advances in biological experimental techniques and future studies may identify this lesion-specific cell adhesion as a major cause of various inflammatory diseases.

## Author contributions

AY, MH, and MF helped to draft the manuscript, AI conceived of this review article and drafted the manuscript.

### Conflict of interest statement

The authors declare that the research was conducted in the absence of any commercial or financial relationships that could be construed as a potential conflict of interest.
